# Supplementary material for: Are carbon emissions trading and green financial instruments synergistic? -Comprehensive quantitative research based on content analysis
Source: PLoS One. 2024 Mar 7;19(3):e0298601. doi: 10.1371/journal.pone.0298601 (PMC10919601; doi:10.1371/journal.pone.0298601)
Supplement: S1 Data — (ZIP) [file pone.0298601.s001.zip › Data/地级市Z_F工作报告.zip!/┴╔─■/┤≤┴1⁄4╩╨/└┤╫╘╣1⁄2╓┌║┼í╛┬φ┐╦╩2╛▌═°í┐_2017.docx]

各位代表：

　　现在，我代表市政府向大会报告工作，请予审议，并请市政协委员提出意见。

　　一、2016年政府工作回顾

　　过去的一年，面对前所未有的严峻复杂形势，在省委、省政府和市委的正确领导下，我们保持定力、沉着应对，全面贯彻党的十八大及十八届三中、四中、五中、六中全会和习近平总书记系列重要讲话精神，坚持五大发展理念和“四个着力”，紧紧把握稳中求进工作总基调，扎实做好稳增长、促改革、调结构、惠民生、防风险各项工作，较好完成市十五届人大四次会议确定的目标任务，实现了“十三五”良好开局。

　　我们着重抓了七件大事：

　　一是抓经济企稳回升。坚持把稳增长作为全年工作的重中之重，全面落实国家和省、市振兴发展系列政策措施，营造良好环境，加强监测调度，千方百计保障要素供给，多措并举促进消费增长、扩大有效投资、稳定外贸出口，经济连续7个季度稳步上升，实现GDP增幅从2%逐季递升至3.5%、3.8%、4.2%、4.3%、5%、5.8%和6.5%。标志着大连经济走出了“低谷”，迈入稳健发展的轨道。

　　二是抓金普新区开放型经济新体制综合试点试验。经申办和争取，金普新区构建开放型经济新体制综合试点试验区获得国务院批准。我们科学制定实施方案、狠抓七大重点试点试验任务落实，全力推进体制机制创新和开放平台建设，新区开发开放步伐明显加快。新引进亿元以上重大项目50多个、总投资700亿元，注册跨境电商802家，经济总量达到2296亿元、增长7%。新区成为引领全市开放发展的强力引擎。

　　三是抓自由贸易试验区申办。把自贸试验区申办作为抢占对外开放制高点的重大举措。经过不懈努力，大连对外开放具有里程碑意义的自贸试验区获批建设。我们制定自贸试验区建设总体方案和“三年行动计划”，瞄准国际通行规则先行先试，复制推广上海等自贸试验区创新制度措施102项，“三互”大通关和国际贸易“单一窗口”走在全国前列。大连又一次站在改革开放的最前沿。

　　四是抓国家自主创新示范区申办和建设。围绕实施创新驱动发展战略，组织力量编制《国家自主创新示范区建设规划》，在省委、省政府支持下，经全力争取，自主创新示范区获得国务院批准。我们主动与先进地区对标，统筹推进自主创新示范区建设，复制实施中关村“6+4”创新政策，在体制创新、成果转化、金融服务、人才引进等方面推出一批重大创新成果和可复制经验，自主创新示范区建设迈出了可喜的步伐。

　　五是抓招商引资。坚持内资与外资并举，引资与引智、引技并重，精心谋划，周密组织，大力开展多种形式的招商引资。全年组织境内外招商12次，引进内资项目215个，总投资3085.4亿元；新增外资项目206个，实际利用外资30.02亿美元、增长11.06%。经济发展后劲持续增强。

　　六是抓城市环境综合整治。下大气力开展污染防治专项行动，累计拆除10吨以下燃煤锅炉604台、淘汰黄标车和老旧车42663辆，全面禁烧秸秆、冥品，严控工地扬尘，空气质量优良率达81.7%，同比提高7个百分点。巩固市容环境综合整治成果，整治行动向铁路沿线、南部海滨及背街小巷延伸，使市容更整洁、环境更优美。

　　七是抓“放管服”改革。把简政放权、放管结合、优化服务作为全面深化改革的核心之要、促进创新创业的关键之举。深化审批制度改革，在全省率先公布权责清单，取消、下放、调整行政职权371项；清理、规范、精简行政审批中介服务事项190项。推进商事制度改革，实施“五证合一、一照一码”制度。创新事中事后监管，全面推开“双随机、一公开”。优化办事流程，有效释放市场活力。

　　中流击水，奋楫者进。这七件大事，事关大连的实力、地位和形象，事关大连发展的机遇、优势和后劲。这七件大事的落实，使大连发展的基础更牢、动力更足、活力更强！

　　2016年，我们统筹兼顾、协调发力，主要做了以下几个方面的工作：

　　（一）推动经济运行稳中向好。

　　主要指标平稳增长。预计实现地区生产总值8150亿元、增长6.5%。一般公共预算收入611.9亿元，增长5.5%，按可比口径增长10%。实现社会消费品零售总额3396亿元，增长10%。就业、物价保持稳定。万元GDP综合能耗下降3.5%。经济向好的基础更加牢固。

　　消费动力不断增强。开展消费升级专项行动，拓展六大领域消费需求，个性化、多样化、便利化消费成为主流。电子商务加快发展，大宗商品电子平台交易额增长6倍多。接待国内外游客7738.8万人次，旅游业综合收入1135亿元、增长12.5%。商品房销售面积、销售额分别增长11%和16.3%。消费对经济增长的贡献率提升，拉动经济增长的作用更加突出。

　　投资结构持续优化。大力推进补短板、调结构、惠民生等重大项目建设，全年开复工亿元以上重大项目660个，完成投资1176.4亿元。高技术产业投资增长36.3%，服务业投资占比达72.5%。积极吸引社会资本参与基础设施建设，推出PPP项目28个。

　　外贸优势逐步增强。着力培育外贸主体、拓展新兴市场、创新业态品牌，新建外贸出口孵化基地9个，新批省级外贸出口基地3个，新创省级出口品牌5 个，新增外贸企业1700家。预计全年完成外贸出口1614亿元，占全省外贸出口总额的56%。我市获批汽车平行进口试点城市。

　　（二）推动产业结构优化升级。

　　传统产业竞争力不断增强。加快制造业向中高端迈进，数控机床、轨道交通、核电装备等领域推出一批新产品、新技术，全市高技术产业增加值增长19.3%，快于规模以上工业11.9个百分点。恒力、固特异、远东工具二期等项目加快建设，大众一汽发动机三期项目顺利投产，东风日产生产整车16.4万辆、增长67%。规模以上工业增加值增长7.4%，高于全国水平1.4个百分点。瓦房店获国家高端装备制造业标准化试点。

　　战略性新兴产业持续壮大。实施战略性新兴产业培育专项行动，国内首个大容量化学储能调峰电站示范项目开工建设，普湾储能装备产业基地一期建成投产，松下汽车动力电池项目即将竣工。英特尔非易失性存储器产品下线，国家布局我市的宇宙半导体项目开工，集成电路产业链日趋完善。大连智能制造产业园、3D打印等项目加快推进。“互联网+”迅猛发展，新技术、新产业、新业态、新模式快速成长，软件和信息服务业实现营业收入1567亿元。战略性新兴产业增加值增长18%，成为未来经济发展的支撑力量。

　　现代服务业发展势头强劲。坚持不懈推动“四个中心”建设，带动服务业向高端化转型。航运中心、物流中心功能显著增强，海港货物吞吐量4.37亿吨、集装箱吞吐量958.3万标箱，空港旅客吞吐量1525万人次。贸易中心建设扎实推进，13个跨境电商线下园区和30家本土线上平台建成启用，大商集团实现销售额2380亿元。金融中心功能增强，新设各类金融及融资机构32家，新增“新三板”“四板”挂牌企业87家，直接融资额增长20.9%；跨境人民币结算额增长19.4%；大商所期货交易量、交易额分别增长37.7%和46.4%。全市服务业增加值增长6.5%，占GDP比重达到51.4%，成为稳增长的“压舱石”。

　　农业发展方式加快转变。稳步推进农村改革，完成380万亩土地确权颁证，实现9万亩土地流转，新建都市型现代农业园区15个，发展设施农业5万亩、海洋牧场10万亩。“互联网+现代农业”发展迅速，全市农业新品种、新技术推广率分别达到99%和87%。农业实现全面丰收，种植业、渔业产量分别增长13.9%和4.5%，粮食产量达30亿斤、增长61%。旅顺成为首个国家级出口农产品质量安全示范区。

　　（三）推动创新创业深入实施。

　　“双创”活力持续增强。制定和实施《国家自主创新示范区建设三年行动计划》，引导企业、高校和科研机构加大科技创新力度，全年发明专利申请量增长38.9%，万人有效发明专利拥有量14.2件，一批发明专利获国家金奖和科技部奖励，新认定高新技术企业144家。深入推进大众创业、万众创新，新登记各类企业32313户，注册资本3493.5亿元，分别增长16.8%和94.4%，每天有近百家企业落地。

　　创新服务体系进一步完善。积极构建产学研用互促互动机制，与高校和科研院所合力推动国家实验室等重大科技基础设施建设。完善“科技指南针”综合服务功能，建设集成电路、储能和智能制造等技术平台5个，新增国家备案众创空间26家。实施促进科技成果转化行动计划，全市技术合同成交额140亿元、增长10.8%。

　　人才强市战略全面推进。落实“5+22”人才政策，财政投入2.1亿元，实施“引才、留才、用才支持计划”。全年向软件企业发放社保补贴资金2.36亿元，为15名高层次人才、147名紧缺人才和3377名新毕业大学生发放住房补贴1600多万元、提供住房970套。全市有90人、2个团队入选国家“千人计划”“万人计划”等重点人才项目。

　　（四）推动改革开放实现突破。

　　供给侧结构性改革有效推进。全面落实“三去一降一补”政策，开展化解过剩产能专项行动，水泥、造纸等“十小”企业全部取缔；出台促进房地产市场健康发展政策措施，商品房待售面积下降9.8%；积极稳妥处置各类债务，有效降低企业杠杆率；开展企业清负减费行动，办理出口退税110.7亿元，为小微企业减免税收12亿元，累计为企业减负338.3亿元。

　　重点领域改革不断深化。加快国资国企改革，组建水务、城建、地铁三大集团，强化国有企业资本运营功能，增强国有企业投资发展能力，全年国有企业营业收入增长3%。推进金普新区 “大部制”改革，机构和人员编制均压缩30%以上。普兰店实施撤市建区，新的管理体制基本到位，行政机构和人员编制分别精简29.2%和25.7%。市直机关所属122户企业脱钩改制和公务用车改革顺利完成。

　　民营经济发展环境持续改善。进一步优化政策环境、市场环境、金融环境、创新环境和法治环境，新出台20条政策措施，促进民营经济发展壮大。开展“百千万”帮扶行动，组建15支专家服务队，选派560名机关干部与2033家企业结对帮扶。建立政策性担保机制，推动银企对接，新增小微企业贷款余额165.3亿元。

　　对外开放领域进一步扩大。加快先导区开发开放步伐，加强对各类园区开放政策、要素保障、物流体系、招商力度和项目服务的监督评估，优化投资环境。长兴岛、花园口等重点开发园区基础设施进一步完善，产业项目不断聚集，成为我市经济转型发展新支撑点。积极参与“一带一路”建设和国际产能合作，国际大通道建设进展顺利，“辽满欧”过境集装箱班列常态化运营，“三星专列”、大连至俄罗斯等5条国际班列开通，全国首列冷链集装箱国际班列在我市投入运营，多式联运入选国家首批示范项目。

　　（五）推动城乡建设取得实效。

　　全域城市化有序推进。开展国家新型城镇化综合试点，推进卫星城和中心镇协调发展，11个中心镇完成基础设施建设投资9.9亿元，皮杨新城纳入国家中小城市综合改革试点。

　　基础设施建设步伐加快。疏港路拓宽改造、渤海大道、旅顺中部通道等工程有序推进；金普城际铁路主体工程、地铁1号线和2号线二期工程、202轨道延伸线等重点交通建设项目即将完工。主城区新增30万个停车泊位、1000个充电桩。开展生活垃圾分类试点，全面启动大连湾、梭鱼湾等10座污水处理厂建设改造和黑臭水体整治工程。主城区新建5座山体健身公园，规划面积达270公顷，健身路径总长达21.8公里。庄河被纳入国家“海绵城市”建设试点。

　　城乡管理水平全面提升。持续开展市容环境综合整治，主城区占道经营、无证营运基本取缔，违法违规建设得到有效遏制，大连火车站和大连北站周边环境整治取得明显成效。美丽乡村建设持续推进，治理水土流失面积20万亩，完成荒山造林3万亩、植树1962万株。智慧城市建设取得新进展，光纤入户改造全面完成，4G网络实现全覆盖。

　　城市文化事业繁荣发展。社会主义核心价值观深入人心，新增主题教育场所35处。“书香大连”等公共文化活动广泛开展，创新政府购买文化服务模式，开展文化艺术讲座、培训800场，推出公益演出400场，专业院团演出1145场，西岗区被评为“中国曲艺名城”。文化产业发展势头良好。

　　社会治理创新深入推进。深化“平安大连”建设，打造社会治安防控体系建设升级版，各类刑事案件下降16.85%。安全生产工作持续加强，事故起数和死亡人数分别下降16.5％和15.3％，近20年来首次实现全年无较大及以上事故发生。扎实推进国家食品安全示范城市创建。社区服务体系不断完善，服务功能持续增强。完善社会矛盾纠纷化解机制，整合12345市民服务网络平台，及时听取和回应人民群众的诉求。圆满完成村委会换届选举工作。我市再获全国“双拥模范城”光荣称号。

　　（六）推动民生福祉持续改善。

　　民生投入持续加大。全年投入民生资金633.7亿元，占财政总支出70%以上。棚户区改造开工8190套，完成“暖房子”工程300万平方米，惠及居民4.6万户。修缮老旧住宅5.8万平方米，改造供热管网453公里、供水管网1万户。改造农村电网1589公里，新建通屯油路840公里。年初承诺的18项重点民生工程全部高质量完成。

　　就业水平稳步提高。大力促进充分就业，以创业带动就业，扶持创业带头人2670人，创业带动就业1.8万人。城镇新增就业10.2万人，稳定就业率达70%以上。本市生源高校毕业生就业率达99.6%，城镇登记失业率控制在2.91%。城乡居民可支配收入达到38220元和15760元，分别增长6.5%和7.5%，城乡群众生活持续改善。

　　社会保障日趋完善。企业退休人员养老金平均达到每月2400元，城乡居民医保财政补助标准达510元，城乡低保标准提高到每人每月640元和410元，失业人员领取失业保险金最高可达每月1224元。完成残疾人家庭无障碍改造、房屋维修1996户，困难残疾人生活补贴标准提高到每人每月70元。建成社区养老服务示范中心14所。

　　社会事业全面发展。新增普惠性幼儿园103所，基本满足幼儿入园需求。组建区域性义务教育集团17个，庄河等4个地区通过国家义务教育发展均衡县评审。在连高校与城市互动发展，职业教育质量不断提升。深化医药卫生体制改革，深入实施“三医联动”“三名工程”，持续提升医疗技术和服务水平。县级公立医院改革全面展开，完成村卫生室标准化建设548所，我市成为全国首批医养结合试点城市。广泛开展全民健身活动，参与国际马拉松和国际徒步大会人数再创新高。65所学校被评为全国校园足球特色学校，大连女足夺得女超联赛冠军。

　　精准扶贫扎实开展。以“七个一”为抓手的扶贫工程全面实施，对128个低收入村、1.9万困难户、40.2万贫困人口实施精准扶贫。设立困难群众家庭台账，派遣工作组驻村入户，实施产业扶贫、教育扶贫、对口扶贫、兜底扶贫，落实116项政策措施，低收入村居民人均可支配收入同比增长9%，高于全市农村居民人均收入增幅1.5个百分点。

　　民族、宗教、外事、侨务、对台、人防、档案、气象、仲裁、防震减灾、民兵预备役、边海防等各项工作都取得了新成绩。

　　（七）推动政府建设全面加强。

　　依法行政深入推进。把法治政府建设摆在更加重要位置，完善依法决策机制，严格履行行政决策法定程序。坚持法无授权不可为、法定职责必须为。大力推进政务公开，该公开的事项全部向社会公开。规范行政执法行为，做到严格、规范、公正、文明执法。认真执行市人大及其常委会的决议、决定，自觉接受人大法律监督和工作监督、政协民主监督、社会舆论监督，认真听取民主党派、工商联、无党派人士和人民团体意见。积极回应人大代表、政协委员的关切，634件人大代表意见、建议和599件政协提案全部办结。

　　工作作风加快转变。深入开展“两学一做”学习教育，严格落实全面从严治党主体责任，大力倡导敢于担当精神和“马上就办”作风，认真解决少数干部庸政懒政怠政等突出问题，政府执行力和行政效能进一步增强。

　　反腐倡廉持续强化。认真贯彻廉洁从政各项要求，自觉践行“五项承诺”，坚决落实中央八项规定精神，有效遏制“四风”反弹。完善政府预算制度，严格“三公”经费预算管理，强化审计监督。全面做实经济数据，开展建设领域腐败问题专项整治，严肃查处损害群众利益的各类案件，政风行风持续好转。

　　各位代表，全市经济社会发展取得的成绩来之不易，是党中央、国务院和省委、省政府及市委坚强领导、大力支持的结果，凝结着全市广大干部群众的智慧和汗水。在此，向给予政府工作大力支持的人大代表和政协委员，向各民主党派、工商联和无党派人士，向离退休老同志，向中省直各部门和兄弟省市驻连机构，向驻连部队和武警官兵，向关心支持大连振兴发展的海内外朋友们表示衷心感谢！

　　在肯定成绩的同时，我们也清醒地看到存在的问题：一是经济发展的体制机制不活，改革的系统性、整体性、协同性有待加强，投资、营商、人才等环境亟待改善。二是产业、产品结构不适应市场变化，产能过剩和需求结构升级矛盾突出。三是有效需求增长乏力，社会投资连续下滑，固定资产投资下降幅度达68.5%，对经济增长的拉动力减弱。四是实体经济困难较多，企业创新和盈利能力不强，经济增长内生动力不足。五是民生领域存在短板，教育、医疗、住房、交通、环保等方面问题亟待解决。六是干部队伍中仍然存在不作为、不担当和作风不深入、不扎实的现象。这些问题必须引起高度重视，并在今后工作中切实加以解决。

　　二、2017年预期目标和主要任务

　　2017年是实施“十三五”规划的重要一年，是供给侧结构性改革的深化之年，也是本届政府履职的收官之年。特别是党的十九大将要召开，对经济社会发展提出更高的要求。

　　今年政府工作的指导思想是：全面贯彻党的十八大及十八届三中、四中、五中、六中全会和习近平总书记系列重要讲话精神，深入落实党中央、国务院全面振兴东北的系列政策措施，统筹推进“五位一体”总体布局和协调推进“四个全面”战略布局，按照中央经济工作会议和省、市十二次党代会的部署，持之以恒落实五大发展理念和“四个着力”，坚持稳中求进工作总基调，以提高发展质量和效益为中心，以深化供给侧结构性改革为主线，以振兴实体经济为重点，以增进民生福祉为出发点和落脚点，更加注重依靠改革开放创新，大力推进“四个中心、一个聚集区”建设，为实现“两先区”的宏伟目标而努力奋斗。

　　经济社会发展的主要预期目标是：地区生产总值增长7%，固定资产投资增长15%，社会消费品零售总额增长10%以上，规模以上工业增加值增长7%，外贸出口增长5%，一般公共预算收入增长5%，城乡居民人均可支配收入增长7%左右，万元GDP综合能耗下降3.2%，居民消费价格涨幅控制在3%左右，城镇登记失业率控制在3%以下。

　　（一）坚持把稳增长放在首位，着力保持经济向好态势。

　　持续加大有效投资。启动建设一批重大产业项目，加快推进恒力石化、松下电池、光洋数控、联通大数据中心等重大项目建设，全年推进亿元以上项目690个，总投资1.6万亿元，当年完成投资1480亿元。加大招商力度，突出高端制造业、现代服务业引资重点，着力引进跨国公司、功能性总部，实际利用外资增长8%以上，引进内资900亿元以上。深化投融资体制改革，重点推进17个PPP项目。

　　全面释放消费潜力。开展“增品种、提品质、创品牌”专项行动，构建“全域旅游”发展格局和“大旅游”工作机制，完善旅游服务设施，从供需两端发力推动旅游业加快发展，深度开发海岛、温泉、健康、休闲等旅游产品，启动老虎滩虎鲸表演馆和石灰石矿万达旅游城建设。统筹发展文化、旅游、养老、健康、体育五大幸福产业，推进服务、信息等六大领域消费工程。加快电子商务与物流快递协同发展，使平台经济和分享经济成为新的增长点。

　　促进外贸优进优出。加快孵化基地、外贸主体和营销网络建设，推动出口产品、技术、服务、品牌创新。促进加工贸易转型，加速提升一般贸易，大力发展服务贸易，建设东北亚服务外包交易中心。加快跨境电商综合试验区建设，发展海运直邮、店铺直购、B2B出口等业务，建设进口商品集散中心。

　　保障经济平稳运行。落实国家和省、市支持企业发展的各项政策措施，加强经济监测预警和分析调度，深入开展“走企业、解难题”活动，解决企业生产经营、技术改造、降本增效、开拓市场、人才引进、融资贷款等实际问题。加大对实体经济金融信贷支持力度，对有效益、有市场、有竞争力的企业，要满足合理信贷需求；对遇到困难的优质企业，要加大协调力度，积极纾解资金紧张问题。全年完成社会融资2600亿元。

　　（二）深化供给侧结构性改革，切实提高发展质量和效益。

　　着力振兴实体经济。把振兴实体经济作为深化供给侧结构性改革的主要任务，加快构建现代产业体系，克服“脱实向虚”倾向，加大政策支持力度，引导各类要素向实体经济聚集。开展改善产品供给专项行动，推动企业开发适销对路新产品，不断提高供给结构对市场需求的适应性。组织实施产品质量提升工程，加快产品质量安全标准与国际标准接轨。加强品牌建设，创建质量强市。建设军民融合发展示范区。

　　全面推进“三去一降一补”。运用市场化、法治化方式，严格执行环保、能耗、质量、安全等法规和标准，推进企业兼并重组，化解过剩产能。坚持拓需求与控供给两手发力，强化政策引导，支持城镇居民改善性住房需求，规范发展房屋租赁市场，逐步消化房地产库存，严格控制房地产土地供应。把去杠杆、防风险摆在更加重要位置，加大股权融资力度，适当扩大直接融资规模，支持企业债转股，降低企业杠杆率。开展降本增效专项行动，加大减税、降费力度，努力降低要素成本、制度成本、物流成本。着力补齐短板，进一步增强公共服务能力，搭建企业创新公共服务平台，支持企业技术创新，完善促进企业发展的基础设施。

　　加快传统产业转型升级。深入落实《中国制造2025大连行动计划》，提高智能制造、绿色制造、精益制造和服务型制造能力，鼓励重点建设工程优先采用地产装备。积极开拓国际市场，推动大连制造与国际产能合作。促进石化、船舶、汽车、轨道交通装备、电子信息等优势产业向全产业链发展，壮大产业集群。加快长兴岛石化产业基地建设，推进恒力炼化一体化项目，发展精细化工产业，打造中国“催化剂之都”。

　　做大做强战略性新兴产业。把培育战略性新兴产业摆在更加突出位置，制定和实施《战略性新兴产业培育和发展三年行动计划》，强力推动高端装备、航空航天、生物医药、新材料、清洁能源、新能源汽车、环保产业等发展。加快集成电路、储能装备、海洋工程等产业实现规模扩张和技术突破；加大对信息产业发展和信息基础设施建设的支持力度，推动软件与信息技术服务业扩量提质；大力发展基于“互联网+”的新技术、新产业、新业态、新模式，推进制造业“双创”平台建设，吸引知名互联网企业深度参与我市电子商务发展，支持互联网就业服务机构实施促进就业创业专项行动。培育壮大海洋新兴产业和海洋服务业，加快建设现代海洋经济示范区。

　　推动现代服务业高效发展。加快航运中心建设，促进港口城市产业一体化、航运交易金融一体化、物流物联集疏一体化，推动大连港向质量效益港、商务便利港、物流枢纽港、绿色低碳港、平安和谐港转型升级。完善物流中心建设规划和网络布局，推进物流园区、物流通道和信息平台建设，优化综合运输体系。完善贸易中心市场体系，加速贸易服务平台建设，培育和引进贸易型总部企业。提升区域性金融中心功能，改善银行、保险、证券、期货等各类金融业务的发展环境，创新金融服务，防范金融风险。吸引国内外金融集团设立区域性机构，加大民营银行和地方金融资产管理公司等筹设力度。办好软交会、专交会、服博会，做大会展经济。

　　提升农业现代化水平。优化农业产业体系、经营体系，大力推广农业共营制模式，培育农村土地股份合作社30家、农民专业合作社50家、家庭示范农场20个，壮大现代农业园区和龙头企业，提高农业全产业链效益。强化农产品标准化生产、品牌创建和质量安全监管，增加绿色优质农产品供给。推进“三权分置”改革，落实农村土地集体所有权、稳定农户承包权、放活土地经营权，加快农村土地确权颁证和土地流转。深化供销合作社改革，加速为农服务中心、互联网农产品销售平台和农产品批发市场建设。搞好第三次全国农业普查。

　　（三）发挥自贸试验区引领作用，提升开放型经济水平。

　　全力推进自贸试验区建设。加快实施自贸试验区总体方案，重点在投资自由化、贸易便利化、金融国际化、管理现代化等方面形成一批具有大连特色的引领性制度成果。建立“1+N”联动机制，加快复制40条创新政策。发挥自贸试验区与自主创新示范区优势叠加效应，构建“双自联动”发展格局，促进制度创新、开放创新、金融创新和科技创新，打造开放和创新融合发展新高地。

　　完善开放型经济新体制。加快金普新区开放型经济新体制综合试点试验区建设，深化体制机制改革，充分发挥引领带动作用。进一步完善长兴岛、花园口、旅顺开发区、中日韩循环经济示范园区的基础设施，出台优惠政策，优化投资环境，有针对性地开展招商引资，使各开放先导区真正成为我市经济发展的先行区。对接国际贸易规则，完善负面清单管理，加强国际贸易“单一窗口”建设，实施海关特殊监管区货物分类监管，推行“联合查验、一次放行”通关新模式。

　　开创对内对外开放新格局。加强与“一带一路”沿线国家产能合作，带动装备、技术、标准、服务“走出去”。积极开展与上海对口合作，在“四个中心”推进、自贸试验区建设、产业发展、政务服务、干部交流和人才培训等领域取得实质性进展。主动对接京津冀协同发展战略。办好夏季达沃斯年会。

　　（四）突出自主创新示范区建设，推动“双创”蓬勃发展。

　　高标准建设自主创新示范区。落实《国家自主创新示范区建设实施意见》和“三年行动计划”，以高新区、金普新区、生态科技创新城为创新平台，落实中关村“6+4”创新政策，开展科创企业投贷联动等金融政策试点，着力推进体制、管理、技术和模式创新，加快区域性科技创新中心建设。

　　完善科技创新服务体系。持续提升“科技指南针”综合服务功能，进一步促进产学研用紧密结合，构建全链条创新创业孵化体系。创建10个高端产业技术创新联盟和60个创新研发平台，推进能源国家实验室、“大连光源”和产业技术研究院、中科院能源学院建设。构建区域科技金融服务和技术交易平台，推进国家技术转移和科技成果转化基地建设。全面加强知识产权保护。

　　推进关键核心技术创新。围绕产业链完善政策链、配置创新链、提升价值链，加快建设智能装备、航空航天、新材料、清洁能源等技术创新中心。推进金普新区智能制造、通用航空、海洋装备3个特色产业园和高新区仿真、车联、健康、电商“四朵云”建设，创建大数据综合试验区。

　　完善“双创”体制机制。构建政府扶持、科技投入、技术转化、产权交易、经费管理、成果评价六大创新机制，完善科技创新政策，设立科技创新基金和科技创新重大专项。着力打造“四众”平台，开展“创业大连，梦响滨城”系列创业活动。完善人才政策体系，下大气力引进人才、用好人才，为有志创新创业者提供梦想成真的舞台。

　　（五）狠抓重点领域改革，全面激发市场活力。

　　全力打造国际化营商环境。把软环境建设摆上重要日程，开展优化投资营商环境专项行动，重拳整治“潜规则”、效率低下等各种制约发展的“顽疾”，严格督查和问责，积极营造市场化、法治化、国际化营商环境。在先导区试行企业投资项目承诺制，探索创新以政策性条件引导、企业信用承诺、监管有效约束为核心的管理模式，试行市场准入负面清单制度。加快“法治大连”“诚信大连”建设，完善政府守信践诺机制，争创全国社会信用体系建设示范城市，构建开放包容、互利合作、亲商安商的良好环境。

　　强化“放管服”改革。全面对标国内先进地区和国际惯例，加快转变政府职能，进一步推进简政放权、放管结合、优化服务改革，深化“一个窗口受理、一站式办理、一条龙服务”，简化流程，提高效率。加快商事制度改革，推进“多证合一”“证照分离”。强化事中事后监管，推动“双随机、一公开”监管全覆盖。推进“互联网+政务服务”，深化电子营业执照管理和全程电子登记改革，变“群众跑腿”为“信息跑路”，变“企业四处找”为“部门协同办”，最大程度方便群众和企业。

　　深化国资国企改革。加快国资监管由管资产向管资本转变。组建国有资本投资运营公司，推动国有企业联合重组，有序转让部分国有企业股权。推动市属国有企业发展混合所有制，与央企合作共建产业园区和产业项目。认真落实中央国企党建工作会议精神，完善国有企业法人治理结构，建立灵活高效的市场化经营机制。完成国企“三供一业”分离移交工作。组建检验检测、国有房屋经营、文化产业三大企业集团，提升国有企业经营效益和国有资本运营效率。

　　加快事业单位分类改革。认真落实中央关于分类推进事业单位改革的指导意见，积极推进经营类事业单位转企改制，依法赋予法人财产权和经营自主权，充分释放事业单位发展活力。对公益类事业单位进行优化整合，减少机构，精简编制。

　　毫不动摇支持民营经济发展壮大。落实支持民营经济发展各项政策，放宽民营经济市场准入，消除各种隐性壁垒，促进民营经济公平参与市场竞争。支持民营企业和社会资本参与国有企业改组改制。健全融资担保体系，推动“银政企保”合作，完善中小微企业贷款融资担保体系。深入实施“育龙计划”，促进民营经济“万木丛生”、茁壮成长。

　　（六）聚焦品质立市战略，高水准实施城乡建设和管理。

　　创新城市发展思路。尊重城市发展规律，统筹空间、规模、产业三大结构，增强城市谋划的全局性；统筹规划、建设、管理三大环节，增强城市建设的系统性；统筹改革、科技、文化三大动力，增强城市发展的持续性；统筹生产、生活、生态三大布局，增强城市功能的宜居性；统筹政府、社会、市民三大主体，增强城市治理的积极性。贯彻“适用、经济、绿色、美观”的建设方针，着力转变城市发展方式、塑造城市特色风貌、提升城市环境质量、创新城市管理服务。

　　依法规划建设城市。增强规划的强制性，推进“多规合一”，坚持一张蓝图绘到底。提高城市设计水平，加强建筑设计管理，保持历史文化风貌。落实工程质量责任，加强建筑安全监管，发展新型装配式建筑，推广建筑节能技术，全面提升城市建筑水平。

　　推进城市精细化管理。按照治住、治好、治久的要求，不断提升市容环境综合整治水平。加强城市交通科学化管理，完善城市交通智能引导系统，提高道路通行能力。进一步提升城市的绿化、亮化水平，给市民提供更加舒适、优美的生活环境。深化城市管理体制改革，推进执法重心下移，健全城市基层治理机制，形成多方参与、多元共治的城市治理格局。

　　完善功能性基础设施。加快推进新机场、大连湾海底隧道、跨海大桥、旅顺中部通道和地铁4号线、5号线、7号线工程建设，完成渤海大道一期、疏港路拓宽改造、河口交通枢纽站工程，金普城际铁路全线贯通，开工建设大连北站综合客运枢纽。加快东港、梭鱼湾、凌水湾和新机场商务区开发建设。推进中心城区垃圾焚烧发电厂二期工程，加快“海绵城市”建设。实施植物园、付家庄海滨、星海公园升级改造工程。启动域外引水工程，彻底解决城市用水后顾之忧。

　　加快智慧城市建设。加速电子政务云中心建设，整合资源，打通“信息孤岛”，推进基础数据库建设。加快全光纤网络城市建设和无线宽带网络建设，优化交通、教育、卫生、城管、口岸、旅游等领域智慧应用系统，迈进“指尖上的城市服务时代”。

　　推进美丽乡村建设。结合农村土地合作社发展，盘活农村闲置建设用地资源，选择有条件的村屯进行宅基地有偿退出和置换试点，引导村民集中居住。突出重点，整治“脏乱差”。新建通屯油路400公里、安全饮水工程15项，实施农村电网改造升级工程，推进信息进村入户和快递物流下乡，在有条件的地区开展光伏暖民示范工程。启动10个“最美村庄”建设试点，加大11个中心镇基础设施、公共服务体系建设力度。创建一批产业特色鲜明、创新要素集聚、产城人文融合、示范效应明显的特色小镇。

　　提高社会治理能力。争创全国首批“雪亮工程”示范城市，提高社会治理的社会化、法治化、智能化、专业化水平。扎实推进遏制重特大事故试点，构建安全风险分级管控和隐患排查双重预防机制。建立食品安全可追溯体系和检验检测体系，加快推进国家食品安全示范城市建设。创新人民群众诉求表达、心理干预、矛盾调处、权益保障机制，及时化解矛盾纠纷。完善社区服务体系，提升基层社区自治能力。

　　（七）推进绿色低碳发展，坚决打好环境保护攻坚战。

　　着力拓展绿色空间。严格落实主体功能区规划，牢牢守住生态红线。大力开展植树造林，新增造林8万亩、植树950万株。实施废旧矿山、裸岩、湿地、滩涂、河流、海岸线等生态修复工程，实现主城区裸露地面全覆盖，新建小区绿化率不低于30%。继续推进主城区山体健身公园建设，完善功能，提升品质。

　　铁腕治理环境污染。全面落实“大气十条”“水十条”“土十条”，持续开展蓝天、碧水、绿地、田园环保行动，强力拆除10吨以下燃煤锅炉，全部取缔黄标车，坚决禁止农村秸杆野外焚烧和城区冥品焚烧，严控工地扬尘。完成环保督查和自查问题治理，城市空气质量优良天数达到300天以上。彻底治理泉水河、春柳河等6条臭水河、57个入海排污口，新建3个、提标改造12个污水处理厂，中心城区污水处理率达到95%以上。全面落实土壤污染防治方案，开展土壤生态修复行动。

　　深入实施节能减排。优化用能结构，推进“煤改电”“煤改气”，市内公交车全部实现清洁能源化。推进清洁取暖，完善生活垃圾分类和再生资源回收体系，推动形成绿色低碳生产方式和生活方式。

　　（八）突出基本民生保障，持续增进人民群众福祉。

　　持续加大民生投入。民之所望，施政所向。着力践行以人民为中心的发展思想，不断增加公共产品和服务供给，高质量完成15项重点民生工程，实施棚户区改造4000套，新增公租房补贴家庭5000户，完成“暖房子”工程230万平方米，维修老旧房屋5万平方米，实施1万户城市居民室内供水管网和2000户弃管楼居民煤气管网改造。组织开展城市民用电梯维修工程，消除安全隐患。让人民群众有更多获得感。

　　强力推进就业增收。完善和落实积极就业政策，加大政府促进就业力度。开展职业技能提升和转岗培训，帮助高校毕业生、就业困难群体就业创业，城镇新增就业8万人，引导更多市民通过创业增加经营性、财产性收入。

　　织密扎牢社会保障网。深化养老保险制度改革，继续提高退休人员养老金待遇水平。理顺城镇居民基本医疗保险和新农合管理体制，城乡居民医保财政补助标准提高到550元。完善社会救助体系，适度提高城乡低保标准。大力发展养老事业，促进医养结合，推广复制居家养老服务“林海模式”。

　　务实推进精准扶贫。以“七个一”为抓手，全面落实精准扶贫、精准脱贫措施，形成产业扶贫、教育扶贫、对口扶贫、兜底扶贫多措并举，政府、企业、社会和扶持对象协同发力的扶贫攻坚局面，确保128个低收入村实现人均可支配收入增长9%以上。

　　（九）大力发展社会事业，促进公共服务均等化。

　　深化教育综合改革。加强学校德育工作，普及中华优秀传统文化教育，扩大义务教育学区化、集团化办学规模，努力办好家门口的每一所学校。发挥优质高中示范作用，推动普通高中优质化、多样化发展。继续推进职业教育资源整合，深化校企合作。鼓励民办学校发展。促进在连高校与城市融合发展，支持一流大学、一流学科建设。

　　推进健康大连建设。坚持公益性导向，深化医药卫生体制改革，促进“三医联动”，建设“三名工程”，完善公立医院考核评价机制，降低药品收入占医疗收入的比重。推进分级诊疗，完善家庭医生签约服务机制，打通社区卫生服务中心与二、三级医院“双向转诊”绿色通道。促进中医药振兴发展，加强重大疾病防控，创建覆盖全生命周期的健康管理服务体系。落实全面两孩政策，强化计划生育服务管理。

　　加快打造文化强市。以社会主义核心价值观为引领，提高文化供给能力和服务质量，推进文化繁荣发展。提高城市文明程度和市民文明素质，深化城市精神宣传教育，开展“最美市民”系列评选活动，在全社会营造崇德向善浓厚氛围。增强公共文化服务能力，完成大连历史博物馆和普湾图书馆、科技馆建设，完善文化设施运行管理和政府购买公益性文化服务机制，推动社区文化活动中心社会化管理。加快文化产业发展，推动文化与科技、教育、金融等深度融合，支持网络视听、动漫游戏、创意设计等新兴文化产业发展。推动文化创新和文艺创作，加快新型媒体发展，深化文艺院团改革，设立名家工作室，实施文艺创作新品、精品扶持计划。发展网络文艺，推出更多人民群众喜闻乐见的优秀作品。

　　促进体育事业和产业协调发展。开展全民健身运动，大力发展竞技体育，实施青少年体育活动促进计划，办好国际马拉松和国际徒步大会等活动。完成青少年足球训练基地建设，加快振兴大连足球。

　　全面实施妇女儿童发展规划，继续做好民族、宗教、外事、侨务、对台、人防、档案、气象、仲裁、防震减灾、双拥共建、民兵预备役和边海防等各项工作。

　　各位代表，振兴发展的伟业艰巨而繁重，人民群众对美好生活的期待现实而热切。新形势新任务要求各级政府及其工作人员必须以时不我待的紧迫感、夙夜在公的使命感和“功成不必在我”的为政境界，勇于打破常规，善于创新思维，敢于求变突破，强化敢于担当精神和“马上就办”作风，严格依法履职，诚心为民办事，通过一项项任务的完成、一个个工程的实施、一件件实事的落实，赢得人民群众的支持和拥护。

　　加强政府廉洁建设，坚定不移反对腐败。更加注重源头治理，严格落实“一岗双责”，建立健全廉政建设与业务工作融合机制，完善行政权力内部流程管控制度，紧紧抓住公共权力运行、公共资源配置、公共资金分配等重点领域，切实做到严肃教育、严明纪律、严格管理、严惩腐败。认真执行《准则》和《条例》，自觉践行“五项承诺”，坚决贯彻中央八项规定精神，严防“四风”反弹，进一步修复政治生态。

　　健全实绩考核办法，严格问效问责，着力解决能力不足“不会为”、动力不足“不想为”、担当不足“不敢为”的问题。认真落实人大及其常委会的各项决议、决定，自觉接受人大法律监督和工作监督、政协民主监督、社会舆论监督，认真办理人大代表的意见、建议和政协委员的提案。

　　各位代表！清风扬正气，奋进正当时。让我们更加紧密地团结在以习近平同志为核心的党中央周围，在市委的坚强领导下，提振干事创业的士气、攻坚克难的勇气、开拓创新的锐气、蓬勃向上的朝气，凝心聚力，勤勉尽责，以振兴发展的优异成绩迎接党的十九大胜利召开！
